# Prevalence and incidence of work-related musculoskeletal disorders in secondary industries of 21st century Europe: a systematic review and meta-analysis

**DOI:** 10.1186/s12891-021-04615-9

**Published:** 2021-08-31

**Authors:** Renée Govaerts, Bruno Tassignon, Jo Ghillebert, Ben Serrien, Sander De Bock, Toon Ampe, Ilias El Makrini, Bram Vanderborght, Romain Meeusen, Kevin De Pauw

**Affiliations:** 1 BruBotics, Vrije Universiteit Brussel, Brussels, Belgium; 2 Human Physiology and Sports Physiotherapy Research Group, Vrije Universiteit Brussel, Brussels, Belgium; 3 Strategic Research Program ‘Exercise and the Brain in Health & Disease: the added value of Human-centered Robotics’, Vrije Universiteit Brussel, Brussels, Belgium; 4 Sciensano Research Institute, Brussels, Belgium; 5 Robotics research group, Vrije Universiteit Brussel and Flanders Make, Brussels, Belgium; 6 Robotics research group, Vrije Universiteit Brussel and IMEC, Brussels, Belgium

**Keywords:** Epidemiology, Occupational health, Ergonomics, Musculoskeletal system, Injury, Prevention

## Abstract

**Objective:**

Over the course of the twenty-first century, work-related musculoskeletal disorders are still persisting among blue collar workers. At present, no epidemiological overview exists. Therefore, a systematic review and meta-analysis was performed on the epidemiology of work-related musculoskeletal disorders (WMSD) within Europe’s secondary industries.

**Methods:**

Five databases were screened, yielding 34 studies for the qualitative analysis and 17 for the quantitative analysis. Twelve subgroups of WMSDs were obtained for the meta-analysis by means of predefined inclusion criteria: back (overall), upper back, lower back, neck, shoulder, neck/shoulder, elbow, wrist/hand, leg (overall), hip, knee, and ankle/feet.

**Results:**

The most prevalent WMSDs were located at the back (overall), shoulder/neck, neck, shoulder, lower back and wrist WMSDs with mean 12-month prevalence values of 60, 54, 51, 50, 47, and 42%, respectively. The food industry was in the majority of subgroups the most prominent researched sector and was frequently associated with high prevalence values of WMSDs. Incidence ratios of upper limb WMSDs ranged between 0.04 and 0.26. Incidence ratios could not be calculated for other anatomical regions due to the lack of sufficient articles.

**Conclusion:**

WMSDs are still highly present among blue collar workers. Relatively high prevalence values and low incidence ratios indicate a limited onset of WMSDs with however long-term complaints.

**Supplementary Information:**

The online version contains supplementary material available at 10.1186/s12891-021-04615-9.

## Introduction

Work-related musculoskeletal disorders (WMSDs) are impairments of the musculoskeletal system, primarily caused by the performance of work tasks and the direct environment in which work is carried out [1]. Secondary industries, known for converting raw materials into products for the consumer, comprise several risk factors that contribute to the development of WMSDs [2]. Repetitive movements, awkward postures as well as continuous and excessive use of force might overload the musculoskeletal system, enhancing the risk of developing WMSDs [3]. Furthermore, psychosocial risk factors such as job related stress, lack of support from colleagues or managers, high mental workload and lack of recognition for the work done are supplementary addons in triggering the development of WMSDs in this sector [4].

The consequences of WMSDs impact both social and individual level, and result in an extensive and varied burden of costs [5]. In European industries, work absenteeism is reported in more than 50% of employees affected by WMSDs, which is significantly higher than in workers infected by the influenza virus (10–12%) [1, 6, 7]. Employees suffering from WMSDs are also absent from work for a longer period of time compared to workers with other health problems [1]. Furthermore, WMSDs are responsible for permanent incapacity in 60% of all reported cases [5]. Not surprisingly, the financial costs of WMSDs in Europe are estimated at 240 billion euros, accounting for 2% of the gross domestic product of EU-15 [5]. In addition to the substantial socio-economic impact, the individual employee has to pay a relatively high price as well, with studies reporting a significant decreased quality of life in people suffering from musculoskeletal disorders [8, 9]. Despite these known negative consequences, a clear epidemiological overview of WMSDs in European secondary industries is missing.

Since 2000, strategies to optimize Europe’s industrial activities are constantly explored to ensure recovery from economic crises and to remain a considerable competitor to other continents [10, 11]. Numerous research fields are therefore encouraged to develop strategies for improving overall industrial work. These innovations change the familiar way of industrial work performance (e.g. robots, exoskeletons) and could therefore impact the development of WMSDs due to changed physical and psychosocial demands [12, 13]. However, in order to objectify the impact of these industrial technologies, recent epidemiological data regarding WMSDs in the previous setting, thus without these technological advances, are necessary first.

Due to the increased risk of incurring WMSDs in secondary industries and the detrimental impact of WMSDs in general, as well as the lack of a clear epidemiological overview on WMSDs in secondary industries of twenty-first century Europe, a systematic review and meta-analysis were performed. The aim was to provide an overall insight on the prevalence and incidence of WMSDs in Europe’s secondary industries during the twenty-first century.

## Methods

The review and meta-analysis was developed and reported in accordance with the preferred reporting items for systematic reviews and meta-analyses [14].

### Search strategy

The PubMed, Web of Science, ScienceDirect, Cochrane library and Scopus databases, were searched for eligible articles. The final search of the databases was performed on the eight of March 2021. Three authors (RG, BT, JG) developed the search strategy in accordance with the PECO framework [15] that comprised key-words related to prevalence and incidence numbers (epidemiology OR incidence OR prevalence), industrial work (industry OR industrial worker OR industrial work OR industrial workplace OR industrial task) and musculoskeletal disorders (musculoskeletal pain OR occupational injury OR injury OR musculoskeletal disorder OR musculoskeletal disease OR musculoskeletal complaint OR musculoskeletal pain OR cumulative trauma disorders). No filters were added with the exception of publication year set from 2000 to 2021 to only include articles that researched WMSDs in the twenty-first century. In addition, reference lists of studies included in this review were screened for relevant articles not provided by the initial search strategy. Detailed descriptions for each database are displayed in supplementary figure A.

### Eligibility criteria

Studies were included if they (i) provided prevalence or incidence data, defined in accordance with the definitions of incidence and prevalence provided by the Centers for Disease Control and Prevention (CDC) [16], (ii) focused on manual work in secondary industry (manufacturing), (iii) included countries of the European Union (EU-28), (iv) reported WMSDs that corresponded to an anatomical region (e.g. neck, back, hip, etc.), (v) adopted an observational study design (cross-sectional, cohort, or health surveys), (vi) used validated or non-validated questionnaires and (vii) were published in peer-reviewed journals. Studies were excluded when they (i) did not provide an overall number of WMSDs in secondary industry, (ii) did not make a clear distinction in manual or administrative workers for reported WMSD data, (iii) failed to clearly differentiate between industry sector, (iv) presented prevalence/incidence numbers based on claims or hospital records and (v) were published before the year 2000.

### Study selection

Databases were searched by one author (RG) and articles were imported in the Rayyan web application for duplicate removal and screening [17]. First, articles were screened on title and abstract by one author according to above mentioned and predefined eligibility criteria. Next, remaining articles were screened on full text by two independent researchers (RG and BT). Disagreements were solved through discussion. If consensus could not be accomplished, a third researcher (JG) would take part in the process to make inclusion by majority possible. When full texts could not be found or data of interest for this meta-analysis was missing, authors would be contacted through e-mail to request full text or data.

### Data extraction and risk of bias

One author (RG) extracted the following data of included full texts to answer the research question: study design, type of industry, period of measurement, response rate, demographic characteristics of included participants (age and gender), tools for examination of WMSDs, examiner (e.g. self-report or occupational physician), type of WMSDs (e.g. neck pain or shoulder pain), prevalence and incidence data per WMSD. Occupational physicians were selected as representative for the examiner variable when options between different healthcare professionals were given. Furthermore, self-reporting prevalence data took precedence over physical examination data when studies reported both. Results will be subdivided in a Prevalence section containing a qualitative and quantitative analysis, and an Incidence section limited to a quantitative analysis.

Risk of bias was assessed in accordance with Hoy et al. [18]. They used a risk of bias tool specifically developed for prevalence studies. Risk of bias was verified through ten questions that can be answered with “high risk” or “low risk”. Questions one to four assess the selection and nonresponse bias (external validity), five to nine the measurement bias and ten the bias related to the analysis. When articles provided insufficient information to answer a question a “high risk”- score was assigned to that item. Risk of bias was analyzed by two independent researchers (RG and JG). Consensus was established through discussion.

### Quantitative synthesis

In order to perform a meta-analysis, studies were included if they reported (i) sufficient demographic information regarding sample size and (ii) data for specific anatomical locations i.e. neck, shoulder, elbow, wrist/hand, back or leg WMSDs. Twelve subgroups of WMSDs were formulated: neck, shoulder, shoulder/neck, elbow, wrist/hand, upper back, lower back, back (for studies that did not make a distinction between upper or lower back), hip, knee, ankle/feet, leg (for studies that did not make a distinction between hip, knee or ankle/feet). Pooling of data occurred in several stages. First, overall prevalence percentages or incidence ratios were calculated for studies reporting data related to subgroups of the investigated sample size i.e. age, gender, skill level (e.g. unskilled versus skilled), workload (e.g. low or high) or type of manual workers (e.g. welder, metal worker, other manual workers). Overall prevalence percentages were calculated using following formula where “p” corresponds to prevalence, “n” to sample size and “n_tot”_ to the sum of all sample sizes: [(p_1_ + … + p_x_) (n_1_ + … + n_x_)]/n_tot_. Overall incidence ratios were calculated using following formula where “i” corresponds to incidence cases, “n” to sample size and “y” to the persons-years at risk: (i_1_ + … + i_x_)/ [(n_1_ + … + n_x_) (y_1_ + … + y_x_)]. In order to minimize heterogeneity, no overall prevalence percentages or incidence ratios were calculated for studies that included prevalence periods (e.g. 12-month or 7-day prevalence). Second, standard errors were calculated for each prevalence rate or incidence ratio using following formula with “p” corresponding to either prevalence rate or incidence ratio and “n” to the sample size: sqrt [p (1-p) / n)]. All calculations were performed in Microsoft Excel (version 2002). Third, mean prevalence and incidence values with associated 95% confidence intervals and heterogeneity (I^2^ statistics) per WMSD-subgroup were calculated through the random-effects model of the R software program (version R-4.0.2). I^2^ statistics displays the variation across included studies that is due to heterogeneity rather than chance [19].

## Results

### Study collection

**Fig. 1:**
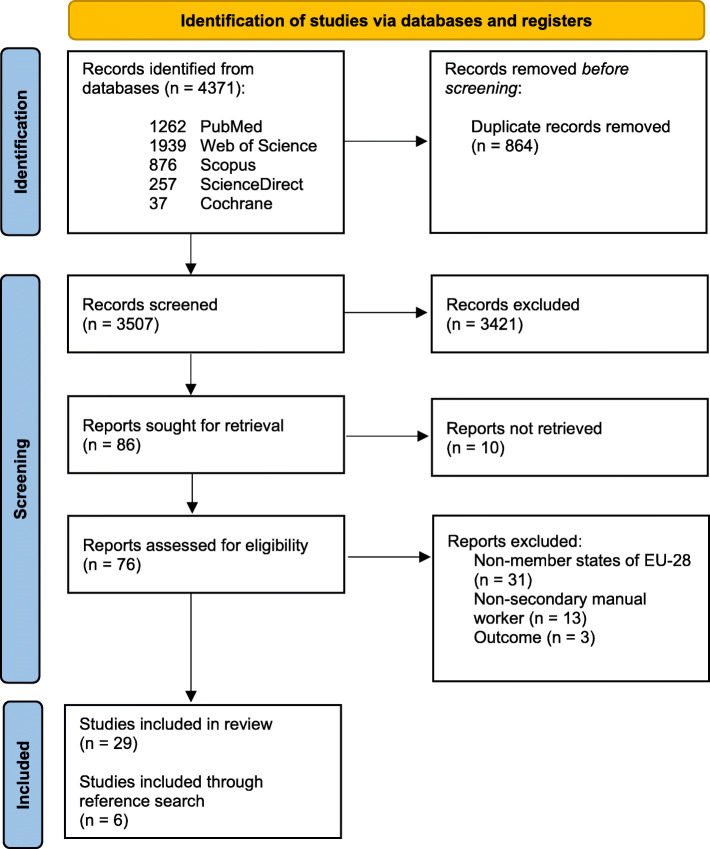
Screening process

A total of 4371 articles were retrieved. After removing duplicates, 3509 articles were subsequently screened by title and abstract. The remaining 88 articles were evaluated on full text as well as six additional articles, obtained from consulting the references of included articles. A total of 35 studies were included in the qualitative analysis of which 24 authors [20–43] discussed prevalence of WMSDs, seven authors [44–50] researched incidence of WMSDs and four authors [51–54] reported both incidence and prevalence rates. Figure 1 displays the results of the screening process in more detail. Due to the limited amount of incidence reports, a meta-analysis could only be performed for studies that reported prevalence data and complied with the predetermined eligibility criteria for quantitative analysis (*n* = 17).

### Risk of bias assessment

Risk of bias assessment resulted in 12 low risk scored studies [20, 22, 25, 31, 32, 34, 39–42, 53, 54], 20 studies with moderate risk scores [21, 23, 24, 26, 28–30, 33, 35–38, 43, 45–49, 51, 52] and 3 studies that received high risk scores [27, 44, 50].

### Prevalence

#### Qualitative analysis

The majority of studies was published between 2000 and 2010 [20, 22–25, 27, 28, 30–33, 36–40, 51, 52, 54] and most included articles were cross-sectionally designed [20–22, 24–26, 29, 31, 32, 34–43, 51, 53]. Thirteen countries and seven different industries were obtained. Further, the majority of studies reported response rates greater than 50% [20–23, 25, 27, 30–34, 37, 38, 40–42, 53, 54] and selected a sample size that ranged between 100 and 500 participants [20, 27, 28, 31–34, 36–38, 40–43, 52–54]. Assessments relying on self-report of the workers were the most prominent tool to evaluate WMSDs [20–24, 30, 31, 35, 37, 38, 40–43]. The Nordic musculoskeletal questionnaire or a modified version was the most utilized questionnaire to identify WMSDs [22, 26, 29, 31, 34, 35, 37, 39, 42, 43, 51] (Table 1).

**Table 1:** Qualitative overview of prevalence of WMSDs in secondary industries of Europe

Author, year, study design, industry & country	Period of measurement	Demographic characteristics	Examination method & response rate	Tools for examination	WMSD & Prevalence
**Aasmoe et al. 2008** [20] Cross-sectional Seafood industry - Norway	December 2000 Prevalence Period: 12 months	T = 744 F = 85 (21%); < 30 years F = 97 (24%); 30–39 years F = 109 (27%); 40–49 years F = 113 (28%); > 50 years M = 119 (35%); < 30 years M = 85 (25%); 30–39 years M = 71 (21%); 40–49 years M = 64 (19%); > 50 years	Self-report 50% of the administrative and production workers	Self-Administered Questionnaire (anonymous): “Have you had any of these symptoms during the last 12 months?”	Neck/Shoulder F = 89%; whitefish Neck/Shoulder F = 88%; shrimp Neck/Shoulder F = 86%; salmon Wrist/Hand F = 62%; whitefish Wrist/Hand F = 66%; shrimp Wrist/Hand F = 82%; salmon Elbow F = 30%; whitefish Elbow F = 38%; shrimp Elbow F = 42%; salmon Back F = 74%; whitefish Back F = 74%; shrimp Back F = 65%; salmon Leg F = 63%; whitefish Leg F = 58%; shrimp Leg F = 61%; salmon Neck/Shoulder M = 71%; whitefish Neck/Shoulder M = 85%; shrimp Neck/Shoulder M = 71%; salmon Wrist/Hand M = 47%; whitefish Wrist/Hand M = 37%; shrimp Wrist/Hand M = 64%; salmon Elbow M = 25%; whitefish Elbow M = 18%; shrimp Elbow M = 24%; salmon Back M = 74%; whitefish Back M = 71%; shrimp Back M = 67%; salmon Leg M = 50%; whitefish Leg M = 48%; shrimp Leg M = 50%; salmon
**Afonso et al. 2014** [21] Cross-sectional Footwear industry - Portugal	January 2013 Prevalence Period: 12 months	F = 66; 22–55 years	Self-report 52.3%	Questionnaire based on 3 validated questionnaires: 1) Nordic Musculoskeletal Questionnaire translated in Portuguese 2) Extended version of the Dutch Musculoskeletal Questionnaire 3) Medium size version of the Copenhagen Psychosocial Questionnaire	Neck T = 32% (*n* = 21) Shoulder T = 23% (*n* = 15) Elbow T = 21% (*n* = 14) Wrist/Hand T = 42% (*n* = 18) Thoracic region T = 6% (*n* = 4) Lumbar region T = 30% (*n* = 20) Hip/Thigh T = 9% (*n* = 6) Knee T = 21% (n = 14) Ankle/Foot T = 15% (*n* = 10)
**Alexopoulos et al. 2006** [22] Cross-sectional Shipyard industry - Greece	November 2003–March 2004 Prevalence Period: 12 months	Blue collar workers = 624; ȳ = 38.7 years	Self-report 98.5% of the white and blue collar workers	Nordic Musculoskeletal Questionnaire translated into Greek	Lower Back = 33%; metal workers Lower Back = 39.8%; welders Lower Back = 37.9%; other blue collar workers Shoulder/Neck = 14.6%; metal workers Shoulder/Neck = 18.3%; welders Shoulder/Neck = 25.2%; other blue collar workers Hand/Wrist = 15.3%; metal workers Hand/Wrist = 10.8%; welders Hand/Wrist = 13.4%; other blue collar workers
**Andersen et al. 2007** [23] Cohort Production industry - Denmark	2-year period Prevalence Period: 12 months	T = 141; skilled workers T = 1874; unskilled workers	Self-report 75%; skilled workers baseline 67%; unskilled workers baseline 79%; skilled workers 24-month follow-up 76%; unskilled workers 24-month follow-up	Self-Administered Questionnaire for regional pain status: “How much have you been bothered by pain during the past 12 months?”	Neck/Shoulder T = 22%; skilled workers baseline Elbow/Forearm/Hand T = 17%; skilled workers baseline Lower Back T = 17%; skilled workers baseline Hip/Knee/Foot T = 12%; skilled workers baseline Neck/Shoulder T = 38%; unskilled workers baseline Elbow/Forearm/Hand T = 22%; unskilled workers baseline Lower Back T = 25%; unskilled workers baseline Hip/Knee/Foot T = 22%; unskilled workers baseline Neck/Shoulder T = 16%; skilled workers 24-month follow-up Elbow/Forearm/Hand T = 8%; skilled workers 24-month follow-up Lower Back T = 19%; skilled workers 24-month follow-up Hip/Knee/Foot T = 17%; skilled workers 24-month follow-up Neck/Shoulder T = 32%; unskilled workers 24-month follow-up Elbow/Forearm/Hand T = 21%; unskilled workers 24-month follow-up Lower Back T = 27%; unskilled workers 24-month follow-up Hip/Knee/Foot T = 24%; unskilled workers 24-month follow-up
**Bang et al. 2005** [24] Cross-sectional Seafood industry – Norway	2001 Prevalence Period: 12 months	T = 1588; m = 39 years F = 889 (56%) M = 699 (44%)	Self-report 49.8% of the industrial and administrative workers	Not further specified questionnaire. Question “Have you, during the last 12 months, felt pain from the neck/shoulders, elbow, wrists/hands, back and legs?”	Neck/Shoulder T = 65.3% Elbow T = 15.3% Wrist/Hand T = 39.7% Back T = 56.1% Leg T = 39.4%
**Bonfiglioli et al. 2006** [25] Cross-sectional Electronic industry - Italy	Not mentioned Prevalence Period: Point prevalence	F = 19 M = 32 ȳ = 36.3 years	Clinician and experienced electrodiagnostic tester 85%	Bilateral median nerve conduction study Physical examination	Carpal Tunnel Syndrome T = 43%
**Claus et al. 2019** [26] Cross-sectional Chemical industry - Germany	January 2011–December 2014 Prevalence Period: 1) 12 months 2) 7 days	T = 1165 (20.9%); < 35 years T = 634 (11.4%); 35–39 years T = 930 (16.7%); 40–44 years T = 1078 (19.4%); 45–49 years T = 1100 (19.8%); 50–54 years T = 664 (11.9%); > 55 years F = 254 (4.6%) M = 5317 (95.4%) ȳ = 43.1 years	Self-report Occupational physicians Not mentioned	Nordic Musculoskeletal Questionnaire (modified) Physical examination	Back T = 66.4%; 12-month prevalence Back T = 26.3%; 7-day prevalence
**De Zwart et al. 2001** [27] **Cross-sectional** **Textile, food, metal, electronic, and production industry - The Netherlands**	1982–1993 Prevalence Period: Point prevalence	F = 80 (30.5%); Textile industry F = 182 (16.0%); Food and beverage industry F = 61 (11.6%); Assemblers of metal products F = 52 (17.0%); Assemblers of electrical products F = 58 (17.4%); Production and related workers NEC M = 182 (69.5%); Textile industry M = 958 (84%); Food and beverage industry M = 463 (88.4%); Assemblers of metal products M = 254 (83.0%); Assemblers of electrical products M = 276 (82.6%); Production and related workers NEC	Self-report Regional occupational health service 70–90%	Self-Administered Questionnaire Physical examination	Neck F = 20.0%; Textile industry Neck F = 23.6%; Food and beverage industry Neck F = 16.4%; Assemblers of metal products Neck F = 30.8%; Assemblers of electrical products Neck F = 5.5%; Production and related workers NEC Shoulder F = 12.5%; Textile industry Shoulder F = 34.1%; Food and beverage industry Shoulder F = 18.0%; Assemblers of metal products Shoulder F = 23.1%; Assemblers of electrical products Shoulder F = 15.5%; Production and related workers NEC Elbow F = 3.8%; Textile industry Elbow F = 3.8%; Food and beverage industry Elbow F = 1.6%; Assemblers of metal products Elbow F = 7.7%; Assemblers of electrical products Elbow F = 1.7%; Production and related workers NEC Wrist F = 1.3%; Textile industry Wrist F = 13.7%; Food and beverage industry Wrist F = 4.9%; Assemblers of metal products Wrist F = 11.5%; Assemblers of electrical products Wrist F = 0%; Production and related workers NEC Neck M = 13.2%; Textile industry Neck M = 10.6%; Food and beverage industry Neck M = 8.6%; Assemblers of metal products Neck M = 1.8%; Assemblers of electrical products Neck M = 11.2%; Production and related workers NEC Shoulder M = 10.4%; Textile industry Shoulder M = 17.2%; Food and beverage industry Shoulder M = 12.5%; Assemblers of metal products Shoulder M = 13.0%; Assemblers of electrical products Shoulder M = 12.7%; Production and related workers NEC Elbow M = 4.4%; Textile industry Elbow M = 4.4%; Food and beverage industry Elbow M = 6.5%; Assemblers of metal products Elbow M = 5.5%; Assemblers of electrical products Elbow M = 3.6%; Production and related workers NEC Wrist M = 5.5%; Textile industry Wrist M = 4.4%; Food and beverage industry Wrist M = 4.3%; Assemblers of metal products Wrist M = 3.9%; Assemblers of electrical products Wrist M = 5.4%; Production and related workers NEC
**Descatha et al. 2003** [28] **Cohort** **Assembly, textile, food, and packaging industry – France**	1993–1994 Prevalence Period: 12 months	T = 479; Assembly industry T = 262; Textile industry T = 307; Food industry T = 160; Packaging industry	Self-report Occupational physicians Not mentioned	Self-Administered Questionnaire Physical examination	Medial epicondylitis T = 5.2%; Assembly industry T = 2.7%; Textile industry T = 5.2%; Food industry T = 2.5%; Packaging industry
**Fouquet et al. 2015** [29] **Cross-sectional** **Agriculture and food, automotive manufacturing, and energy industry - France**	2002–2005 Prevalence Period: 7 days	Not mentioned	Occupational physicians 18% of the occupational physicians (all industries)	Nordic Musculoskeletal Questionnaire	Thoracic spine F = 15.0% (n = 113); Agricultural and food industry F = 19.8% (*n* = 116); Automotive manufacturing industry F = 0% (*n* = 0); Energy M = 10.4% (*n* = 182); Agricultural and food industry M = 19.4% (*n* = 62); Automotive manufacturing industry M = 8.3% (*n* = 12); Energy industry
**Ha et al. 2009** [51] **Cross-sectional** **Food and drink, garment, shoe and leather, manufacture of wood and wood products, manufacture of pulp, paper and paper products, publishing, printing and reproduction of recorded media, chemical, manufacture of rubber and plastic products, manufacture of other non-metallic mineral products, manufacture of basic metals, manufacture of fabricated metal products, manufacture of machinery and equipment not elsewhere classified, manufacture of electrical and optical equipment, manufacture of motor vehicles, manufacture of other transport equipment, manufacture of furniture and wood, recycling industry - France**	April 2002–April 2005 Prevalence Period: 12 months	F = 113; Food and drink industry F = 12; Garment industry F = 28; Shoe and leather industry F = 6; Manufacture of wood and wood products F = 12; Pulp, paper and paper products manufacturing F = 9; Publishing, printing, reproduction of recorded media F = 2; Chemical industry F = 45; Manufacture of rubber and plastic products F = 2; Non-metallic mineral products manufacturing F = 6; Manufacture of basic metals F = 11; Manufacture of fabricated metal products F = 26; Manufacture of electrical and optical equipment NEC F = 69; Manufacture of electrical and optical equipment F = 2; Manufacture of motor vehicles F = 2; Manufacture of other transport equipment F = 45; Manufacture of furniture and wood industries F = 0; Recycling M = 182; Food and drink industry M = 1; Garment industry M = 8; Shoe and leather industry M = 24; Manufacture of wood and wood products M = 52; Pulp, paper and paper products manufacture M = 17; Publishing, printing, reproduction of recorded media M = 8; Chemical industry M = 84; Manufacture of rubber and plastic products M = 22; Non-metallic mineral products manufacturing M = 23; Manufacture of basic metals M = 91; Manufacture of fabricated metal products M = 89; Manufacture of electrical and optical equipment NEC M = 89; Manufacture of electrical and optical equipment M = 63; Manufacture of motor vehicles M = 91; Manufacture of other transport equipment M = 57; Manufacture of furniture and wood industries M = 7; Recycling	Neurologists Occupational physicians 17.4% of the occupational physicians	Nordic Musculoskeletal Questionnaire Physical examination according the “criteria document” (if symptoms had occurred during the last 12 months)	Upper limb musculoskeletal disorder F = 13.3%; Food and drink industry F = 33.3%; Garment industry F = 10.7%; Shoe and leather industry F = 50.0%; Manufacture of wood and wood products F = 33.3%; Pulp, paper and paper products manufacturing F = 11.1%; Publishing, printing and reproduction of recorded media F = 50.0%; Chemical industry F = 33.3%; Manufacture of rubber and plastic products F = 0.0%; Manufacture of non-metallic mineral products F = 50.0%; Manufacture of basic metals F = 18.2%; Manufacture of fabricated metal products F = 23.1%; Manufacture of electrical and optical equipment NEC F = 14.5%; Manufacture of electrical and optical equipment F = 0.0%; Manufacture of motor vehicles F = 50.0%; Manufacture of other transport equipment F = 17.8%; Manufacture of furniture and wood industries F = 0%; Recycling M = 12.1%; Food and drink industry M = 0%; Garment industry M = 0%; Shoe and leather industry M = 12.5%; Manufacture of wood and wood products M = 11.5%; Manufacture of pulp, paper and paper products M = 5.9%; Publishing, printing and reproduction of recorded media M = 0.0%; Chemical industry M = 14.3%; Manufacture of rubber and plastic products M = 4.6%; Non-metallic mineral products manufacturing M = 8.7%; Manufacture of basic metals M = 18.7%; Manufacture of fabricated metal products M = 14.6%; Manufacture of electrical and optical equipment NEC M = 15.7%; Manufacture of electrical and optical equipment M = 25.4%; Manufacture of motor vehicles M = 11.1%; Manufacture of other transport equipment M = 10.5%; Manufacture of furniture and wood industries M = 0%; Recycling
**Harkness et al. 2003** [30] **Cohort** **Shipyard industry - UK**	Not mentioned Prevalence Period: 1 month	T = 82; 12-month follow-up T = 67; 24-month follow-up	Self-report 86%; 12-month follow-up 92%; 24-month follow-up	Questionnaire for pain involving one question: “Thinking back over the past month, have you had any ache or pain which lasted for one day or longer?’ If so, indicate the site of this pain on a line drawing of the body.”	Shoulder T = 11% (*n* = 9); 12-month follow-up Shoulder T = 19% (*n* = 13); 24-month follow-up
**Hussain 2004** [31] **Cross-sectiona** **Transport industry - UK**	Not mentioned Prevalence Period: 12 months	T = 323; ȳ = 36.5 years	Self-report 70%	Modified version of the Nordic Musculoskeletal Questionnaire	Neck T = 60% (*n* = 194) Shoulder T = 57% (*n* = 184) Upper Back T = 17% (*n* = 55) Elbow T = 20% (*n* = 65) Lower Back T = 65% (*n* = 211) Wrist/Hand T = 46% (*n* = 149) Hip T = 8% (*n* = 26) Knee T = 39% (*n* = 126) Ankle/Foot T = 13% (*n* = 42)
**Isolani et al. 2002** [32] **Cross-sectional** **Food industry - Italy**	March–May 1998 Prevalence Period: Point prevalence	T = 114; ȳ = 38.0 years F = 21 (18%); 22–61 years M = 93 (82%); 22–61 years	Trained physicians 71%	Interview Physical examination Median NCSs	Carpal tunnel syndrome T = 53% (*n* = 60)
**Kaergaard and Andersen 2000** [33] **Cohort** **Textile industry - Denmark**	1994–1997 Prevalence Period: Point prevalence	F = 243; ȳ = 38.3 years	Self-report Trained physicians who were blinded to the answers from the questionnaire 94%; baseline 55%; follow-up	Questionnaire about current musculoskeletal complaints Physical examination (neck and arms)	Neck/Shoulder T = 77.6%; baseline Myofascial pain syndrome T = 15.2%; baseline Rotator cuff tendinitis T = 5.8%; baseline
**Le Manac’h et al. 2012** [34] **Cross-sectional** **Food, metal, electronic industry – France**	April 2002–April 2005 Prevalence Period: 12 months	T = 295; Food industry T = 102; Metal industry T = 115; Electronic industry ȳ = 38.7 years	Self-report Occupational physicians 93% of all workers	Nordic Auto-Questionnaire Physical examination, if indicated	Knee bursitis T = 1.4% (n = 4); Food industry Knee bursitis T = 2.0% (n = 2); Metal industry Knee bursitis T = 0.9% (n = 1); Electronic industry
**Leclerc et al. 2001** [52] **Cohort** **Electrical, textile, food, and packaging industry - France**	1993–1994 1996–1997 Prevalence Period: Point prevalence	T = 247; Electrical industry T = 63; Textile industry T = 143; Food industry T = 103; Packaging	Self-report Occupational physicians 42.11%	Self-Administered Questionnaire Physical examination	Carpal tunnel syndrome T = 21.9% (*n* = 54); Electronic industry Carpal tunnel syndrome T = 27.0% (*n* = 17); Textile industry Carpal tunnel syndrome T = 13.3% (*n* = 19); Food industry Carpal tunnel syndrome T = 31.1% (*n* = 32); Packaging Lateral epicondylitis T = 20.2% (*n* = 50); Electronical industry Lateral epicondylitis T = 4.8% (n = 3); Textile industry Lateral epicondylitis T = 7.0% (*n* = 10); Food industry Lateral epicondylitis T = 7.8% (*n* = 8); Packaging Wrist tendinitis T = 8.5 (n = 21); Electronical industry Wrist tendinitis T = 3.2% (*n* = 2); Textile industry Wrist tendinitis T = 21.0% (*n* = 30); Food industry Wrist tendinitis T = 10.7% (*n* = 11); Packaging
**Lima et al. 2019** [35] **Cross-sectional** **Food industry - Portugal**	May 2016 Prevalence Period: 12 months	T = 20 (27%); 24–33 years T = 21 (28%); 34–44 years T = 17 (23%); 44–52 years T = 16 (22%); 54–63 years F = 53 (71.6%) M = 21 (28.4%) ȳ = 41.9 years	Self-report 35.23%	Nordic Musculoskeletal Questionnaire	Neck T = 15% (n = 3); 24–33 years Neck T = 29% (*n* = 6); 34–44 years Neck T = 65% (n = 11); 44–53 years Neck T = 63% (n = 10); 54–63 years Lower Back T = 35% (*n* = 7); 24–33 years Lower Back T = 19% (n = 4); 34–44 years Lower Back T = 35% (n = 6); 44–53 years Lower Back T = 69% (n = 11); 54–63 years Right Shoulder T = 30% (n = 6); 24–33 years Right Shoulder T = 24% (n = 5); 34–44 years Right Shoulder T = 53% (n = 9); 44–53 years Right Shoulder T = 38% (n = 6); 54–63 years
**Nordander et al. 2008** [36] **Cross-sectional** **Manufacturing industry and mechanical assembly industry - Sweden**	Not mentioned Prevalence Period: 7 days	F = 172; ȳ = 42.0 years M = 105; ȳ = 36.0 years	Examiners not specified Not mentioned	Questionnaire-based interview Physical examination (modified scheme by Ohlsson et al.)	Neck/Shoulder F = 61% (*n* = 104) Elbow/Hand F = 55% (*n* = 95) Lower Back F = 30% (*n* = 52) Knee/Foot F = 41% (*n* = 70) Tension neck syndrome F = 20% (*n* = 34) Cervicalgia F = 6% (*n* = 11) Shoulder tendinitis F = 16% (*n* = 27) Acromioclavicular syndrome F = 12% (*n* = 20) Epicondylitis F = 6% (n = 11) Pronator teres syndrome F = 2% (*n* = 3) Radial tunnel syndrome F = 1% (n = 1) Carpal tunnel syndrome F = 8% (n = 13) Overused hand syndrome F = 2% (n = 3) Peritendinitis/Tenosynovitis F = 7% (*n* = 12) De Quervain’s disease F = 1% (n = 2) Neck/Shoulder M = 36% (*n* = 38) Elbow/Hand M = 34% (*n* = 36) Lower Back M = 27% (*n* = 28) Knee/Foot M = 32% (n = 34) Tension neck syndrome M = 12% (n = 13) Cervicalgia M = 3% (n = 3) Shoulder tendinitis M = 6% (*n* = 6) Acromioclavicular syndrome M = 7% (n = 7) Epicondylitis M = 3% (n = 3) Pronator teres syndrome M = 0% (*n* = 0) Radial tunnel syndrome M = 0% (n = 0) Carpal tunnel syndrome M = 2% (n = 2) Overused hand syndrome M = 0% (n = 0) Peritendinitis/Tenosynovitis M = 3% (n = 3) De Quervain’s disease M = 0% (n = 0)
**Ólafsdóttir and Rafnsson 2000** [37] **Cross-sectional** **Fish industry - Iceland**	Not mentioned Prevalence Period: 1) 12 months 2) 7 days	T = 254 F = 49 (19%); 16–19 years F = 80 (32%); 20–29 years F = 125 (49%); 30–54 years	Self-report 71%	Nordic Musculoskeletal Questionnaire	Neck = 69%; 12-month prevalence Shoulder = 78%; 12-month prevalence Elbow = 17%; 12-month prevalence Wrist = 47%; 12-month prevalence Upper Back = 37%; 12-month prevalence Lower Back = 68%; 12-month prevalence Hip = 22%; 12-month prevalence Knee = 28%; 12-month prevalence Ankle = 24%; 12-month prevalence Head = 56%; 12-month prevalence Fingers = 31%; 12-month prevalence Neck = 44%; 7-day prevalence Shoulder = 47%; 7-day prevalence Elbow = 9%; 7-day prevalence Wrist = 26%; 7-day prevalence Upper Back = 18%; 7-day prevalence Lower Back = 39%; 7-day prevalence Hip = 16%; 7-day prevalence Knee = 15%; 7-day prevalence Ankle = 15%; 7-day prevalence Head = 39%; 7-day prevalence Fingers = 19%; 7-day prevalence
**Pope et al. 2001** [38] **Cross-sectional** **Packaging industry - UK**	Not mentioned Prevalence Period: 1 month	T = 203	Self-report 83% of all workers	Manikin and Shoulder Disability Questionnaire	Shoulder T = 27% (*n* = 55)
**Ricco and Signorelli 2017** [53] **Cross-sectional** **Meat industry - Italy**	January 2012 December 2013 Prevalence Period: Point prevalence	T = 434; ȳ = 37.0 years F = 198 (45.6%) M = 236 (54.4%)	Trained clinician 91.8%	Full medical assessment Ultrasonography and/or NCS in clinically possible cases	Carpal tunnel syndrome F = 17.2% (n = 34) Carpal tunnel syndrome M = 11.4% (n = 27)
**Roquelaure et al. 2002** [54] **Cohort** **Shoe industry - France**	1996–1997 Prevalence Period: 12 months	Year 1996: T = 253; ȳ = 40.2 years F = 158 (62%) M = 95 (38%) Year 1997: T = 191; ȳ = 41.1 years F = 117 (61%) M = 74 (39%)	Occupational physicians 90%	Interview Physical examination	Tension neck syndrome T = 7.5% (n = 19); 1996 Rotator cuff syndrome = 7.9% (n = 20); 1996 Medial epicondylitis T = 0% (n = 0); 1996 Lateral epicondylitis T = 2.0% (n = 5); 1996 Cubital tunnel syndrome T = 5.2% (n = 10); 1996 Radial tunnel syndrome T = 0.4% (n = 1); 1996 Carpal tunnel syndrome T = 18.2% (*n* = 46); 1996 Guyon’s canal syndrome T = 0.4% (n = 1); 1996 Hand/Wrist tendinitis T = 2.4% (n = 6); 1996 Tension neck syndrome T = 4.2% (*n* = 8); 1997 Rotator cuff syndrome T = 9.5% (*n* = 18); 1997 Medial epicondylitis T = 0.5% (n = 1); 1997 Lateral epicondylitis T = 3.1% (n = 6); 1997 Cubital tunnel syndrome T = 4.2% (n = 8); 1997 Radial tunnel syndrome T = 1.0% (n = 2); 1997 Carpal tunnel syndrome T = 22.0% (*n* = 42); 1997 Guyon’s canal syndrome T = 0.5% (n = 1); 1997 Hand/Wrist tendinitis T = 3.1% (n = 6); 1997
**Roquelaure et al. 2006** [39] **Cross-sectional** **Food, textile, wood, paper, chemical, steel, machine and equipment, computer, automotive, furniture and wood industry - France**	April to September 2002 May to October 2003 Prevalence Period: Not specified	F = 69; Food industry F = 31; Textile industry F = 5; Wood industry F = 20; Paper industry F = 39; Chemical industry F = 9; Steel industry F = 22; Machine and equipment industry F = 32; Computer industry F = 3; Automotive industry F = 43; Furniture industry F = 112; Food industry F = 112; Textile industry F = 23; Wood industry F = 55; Paper industry F = 74, Chemical industry F = 81; Steel industry F = 72; Machine and equipment industry F = 51; Computer industry F = 40; Automotive industry F = 50; Furniture industry	Occupational physicians 17.4% of the Sentinal network occupational physicians	Nordic Musculoskeletal Questionnaire VAS If symptoms occurred during the past 12 months: physical examination based on the criteria document for the evaluation of work-related upper-limb MSDs	Upper extremity disorders F = 15.9% (n = 11); Food industry F = 19.4% (n = 6); Textile industry F = / (n = 2); Wood industry F = 25.0% (n = 5); Paper industry F = 35.9% (*n* = 14); Chemical industry F = 33.3% (n = 3); Steel industry F = 27.3% (n = 6); Machine and equipment industry F = 12.5% (n = 4); Computer industry F = / (n = 1); Automotive industry F = 20.9% (*n* = 9); Furniture industry M = 12.5% (n = 14); Food industry M = 0% (n = 0); Textile industry M = 13% (n = 3); Wood industry M = 10.9% (n = 6); Paper industry M = 12.2% (n = 9); Chemical industry M = 14.8% (n = 12); Steel industry M = 12.5% (n = 9); Machine and equipment industry M = 7.8% (n = 4); Computer industry M = 20% (n = 8); Automotive industry M = 20.0% (n = 5); Furniture industry
**Sormunen et al. 2009** [40] **Cross-sectional** **Meat and dairy industry - Finland**	1997 Prevalence Period: 12 months	T = 117 F = 46%; ȳ = 35.0 years M = 54%; ȳ = 33.0 years	Self-report 85%	Questionnaire	Neck/Shoulder F = 89% (*n* = 424); 18–64 years Neck/Shoulder F = 90% (*n* = 275); 18–39 years Neck/Shoulder F = 86% (*n* = 149); 49–64 years Shoulder F = 65% (*n* = 291); 18–64 years Shoulder F = 60% (*n* = 178); 18–39 years Shoulder F = 75% (*n* = 113); 49–64 years Lower Back F = 68% (*n* = 303); 18–64 years Lower Back F = 65% (*n* = 193); 18–39 years Lower Back F = 73% (*n* = 109); 49–64 years Wrist F = 71% (*n* = 318); 18–64 years Wrist F = 70% (*n* = 213); 18–39 years Wrist F = 72% (*n* = 105); 49–64 years Neck/Shoulder M = 78% (*n* = 432); 18–64 years Neck/Shoulder M = 75% (n = 318); 18–39 years Neck/Shoulder M = 87% (n = 113); 49–64 years Shoulder M = 57% (*n* = 305); 18–64 years Shoulder M = 50% (*n* = 204); 18–39 years Shoulder M = 82% (*n* = 99); 49–64 years Lower Back M = 64% (*n* = 347); 18–64 years Lower Back M = 60% (*n* = 253); 18–39 years Lower Back M = 75% (*n* = 92); 49–64 years Wrist M = 58% (*n* = 311); 18–64 years Wrist M = 57% (*n* = 237); 18–39 years Wrist M = 63% (*n* = 74); 49–64 years
**Sundstrup et al. 2014** [41] **Cross-sectional** **Meat industry - Denmark**	Not mentioned Prevalence Period: 3 months	T = 595; ȳ = 44.0 years F = 11% M = 89%	Self-report 92%	Questionnaire: 0–10 modified VAS scale for neck, shoulder, elbow, and hand/wrist regions defined by drawings from the Nordic Musculoskeletal Questionnaire.	Neck T = 48% Shoulder T = 60% Elbow T = 40% Hand/Wrist T = 52%
**Wixted et al. 2018** [42] **Cross-sectiona** **Manufacturing industry - Ireland**	Not mentioned Prevalence Period: 12 months	T = 47 (20%); 21–30 years T = 97 (41.3%); 31–40 years T = (27.7%); 41–50 years T = (11.1%); 51–60 years F = 47 (20%) M = 188 (80%)	Self-report 80%	Nordic Musculoskeletal Questionnaire (Amended)	Neck T = 41% (*n* = 96) Shoulder T = 46% (*n* = 108) Upper back T = 28% (*n* = 66) Lower back T = 57% (*n* = 134)
**Weyh et al. 2020** [43] **Cross-sectional** **Steel industry - Germany**	July 2016 to May 2017 Prevalence Period: 12 months	T = 145; ȳ = 35.8 years F = 2 (1%) M = 143 (99%)	Self-report Not mentioned	Modified version of the Nordic Musculoskeletal Questionnaire	Neck T = 61% Shoulder T = 55% Elbow T = 32% Forearm T = 32% Upper back T = 36% Lower back T = 71% Hip = 9% Knee = 44% Lower leg = 16%

*NEC* Not elsewhere classified, *T* Total, *M* Male, *F* Female, *m* Median age, *ȳ* Mean age

Although the preponderance of studies reported prevalence data over a 12-month period [20–24, 28, 31, 35, 40, 42, 43, 51, 54] or discussed point prevalence data [25, 27, 32, 33, 52, 53], prevalence periods varied for other included articles [26, 30, 37–39, 41]. WMSDs were mostly reported in anatomical regions of the neck, shoulder, elbow, hand/wrist, back and legs. Consequently, these anatomical regions are included in the quantitative analysis (3.3.2 Quantitative analysis) and will be discussed in more detail with their corresponding prevalence data (Table 1).

#### Quantitative analysis

Seventeen of the 28 studies were selected based on aforementioned eligibility criteria [20–24, 26, 27, 30, 31, 33, 35–38, 40, 42, 43].

##### 12-month prevalence

Twelve studies [20–24, 26, 31, 35, 37, 40, 42, 43] were divided in 12 subgroups of WMSDs: neck, shoulder, shoulder/neck, elbow, wrist/hand, upper back, lower back, back (for studies that did not make a distinction between upper or lower back), hip, knee, ankle/feet, leg (for studies that did not make a distinction between hip, knee or ankle/feet). The heterogeneity analysis illustrated a considerable heterogeneity for the majority of subgroups (neck, shoulder, shoulder/neck, wrist/hand, back, upper back, lower back, leg and knee) with ranges between 76 and 96%. A substantial heterogeneity was obtained for the hip subgroup (I ^2^ = 63%) and both the elbow and ankle/feet subgroups displayed a heterogeneity of non-importance (I^2^ = 5% and I^2^ = 36%, respectively) [55]. Due to the lack of sufficient articles, additional subgroup analyses could not be performed to further investigate these high heterogeneity values. Pooled sample sizes of 9540, 7464, 1097, 2984, 4507, 5405, 788, 4922, 1023, 5710, 643 and 788 blue-collar secondary industry workers were obtained for back, shoulder/neck, neck, shoulder, lower back, wrist/hand, knee, leg, upper back, elbow, ankle/feet and hip subgroups, respectively. A distinct distribution of WMSDs over time could not be identified, however the food industry was the industry generally most represented in all subgroups. On average, industrial workers had the most WMSDS in the back (back: mean 60%; SD 13%, lower back: mean 47%; SD 20%), shoulder (shoulder: mean 50%; SD 18%, shoulder/neck: mean 54%; SD 27%) and neck regions (mean 51%; SD 15%). Hip complaints were the least prevalent (mean 11%; SD 7%) (Table 2).

**Table 2:** Overall prevalence of WMSDs per anatomical region

*Anatomical location*	*Mean (%) with 95% CI*	*Median (%)*	*Range (%)*	*SD (%)*	*I*^*2*^*(%)*
*Back*	60 [49;70]	66	38–72	13	83
*Shoulder/Neck*	54 [31;74]	51	19–83	27	96
*Neck*	51 [40;62]	51	32–69	15	86
*Shoulder*	50 [37;63]	51	23–78	18	91
*Lower back*	47 [33;61]	47	24–71	20	93
*Wrist/Hand*	42 [31;54]	44	14–64	16	90
*Knee*	33 [24;43]	34	21–46	11	76
*Leg*	29 [18;43]	23	16–56	16	89
*Upper back*	22 [13;37]	28	6–37	12	90
*Elbow*	21 [18;24]	21	15–30	4	5
*Ankle/Feet*	17 [12;23]	15	13–24	6	36
*Hip*	11 [7;18]	9	8–22	7	63

**Fig. 2:**
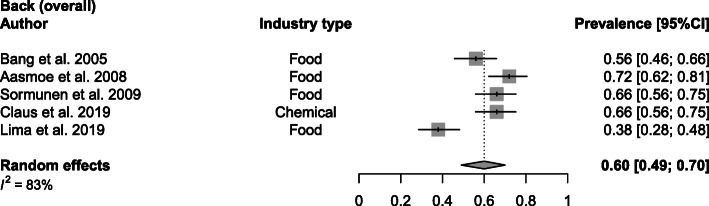
Meta-analytic overview of prevalence of back WMSDs

A mean of 60% (range 38–72%; SD 13%) back WMSDs was reported in secondary industries (Fig. 2). The food industry was the industry most represented in this analysis [20, 24, 35, 40]. Lower back WMSDs obtained a mean prevalence of 47% (range 24–71%; SD 20%) and were researched in the food, transport, shipyard, production, textile, manufacturing and steel industry (Figure B supplementary material). With the exception of the production industry, where Andersen et al. [23] reported both baseline and follow-up prevalence data, all industries were represented equally [21–23, 31, 37, 42, 43]. Upper back WMSDs were prevalent in 22% (range 6–37%; SD 12) of the investigated pooled sample size with food [37], transport [31], textile [21], manufacturing [42] and steel [43] industries included in the subgroup analysis (Figure C supplementary material).

**Fig. 3:**
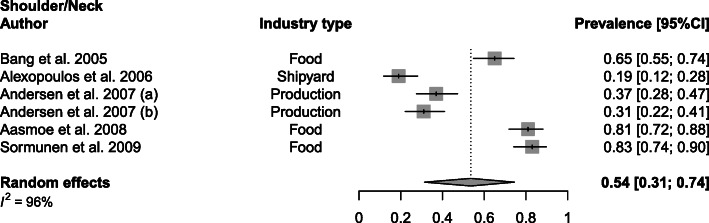
Meta-analytic overview of prevalence of shoulder/neck WMSDs. (a) = baseline; (b) = 24-month follow-up

Shoulder/neck WMSDs obtained a mean prevalence of 54% (range 19–83%; SD 27%) and were reported in the food [20, 24, 40], shipyard [22] and production [23] industry (Fig. 3). Isolated neck WMSDs were prevalent in 51% (range 32–69%, SD 15%) of the secondary industrial workers (Figure D supplementary material) and isolated shoulder WMSDs in 50% (range 23–78%; SD 18%) of the workers researched (Figure E supplementary material). In both subgroups, the food industry was the industry sector most represented [35, 37, 40] and also the transport [31], textile [21], manufacturing [42] and steel [43] industry were included in the analysis.

**Fig. 4:**
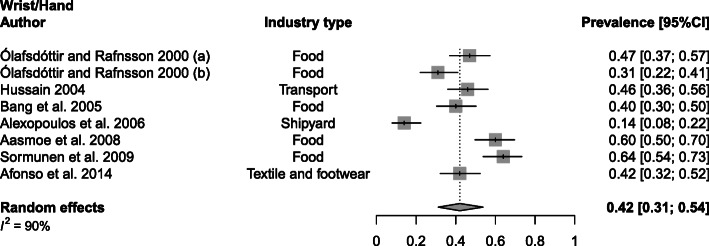
Meta-analytic overview of prevalence of wrist/hand WMSDs. (a) = wrist; (b) = fingers

**Fig. 5:**
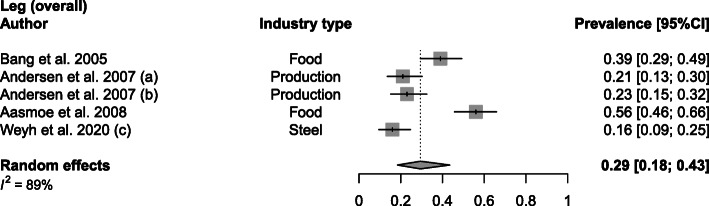
Meta-analytic overview of prevalence of leg WMSDs. (a) = baseline; (b) = 24-month follow-up

Wrist/hand WMSDs, with a mean prevalence of 42% (range 14–64%; SD 16%) were investigated in the food [20, 24, 37, 40], transport [31], shipyard [22] and textile [21] industry (Fig. 4). Further, a prevalence of 29% (range 16–56%; SD 16%) was obtained for overall leg WMSDs in food [20, 24], production [23] and steel industries [43] (Fig. 5). Considering studies that differentiate between hip, knee and ankle/feet WMSDs, mean prevalence values were obtained of 11% (range 8–22; SD 7%), 33% (range 21–46%; SD 11%) and 17% (range 13–24%; SD 6%), respectively (Figure F, G, H supplementary material).

**Fig. 6:**
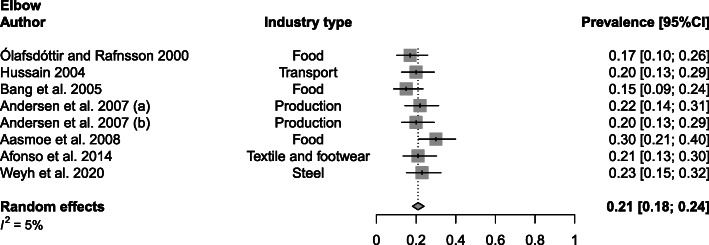
Meta-analytic overview of prevalence of elbow WMSDs. (a) = baseline; (b) = 24-month follow-up

Food [20, 24, 37], transport [31], production [23], textile [21] and steel [43] industries were all affected by elbow WMSDs with a prevalence mean of 21% (range 15–30%; SD 4%) (Fig. 6).

##### 3-month prevalence

Sundstrup et al. [41] reported 3-month prevalence values of 60, 52, 48, 40% for the shoulder, wrist/hand, neck and elbow, respectively. No further analysis was performed due to the lack of sufficient articles.

##### 1-month prevalence

Two studies reported a 1-month prevalence value for shoulder WMSDs with a calculated mean prevalence of 22% (range 11–27%; SD 8%) [30, 38].

##### 7-day prevalence

WMSDs during a 7-day period were researched in three studies [26, 36, 37]. Prevalence values of 47, 44, 26, 18, 16 and 15% for shoulder, neck, wrist/hand, upper back, hip and ankle feet WMSDs, respectively, were solely researched in one study [37]. Overall back WMSDs with a prevalence of 26% were reported by [26] and neck/shoulder WMSDs with a prevalence of 52% were discussed by Nordander et al. [36]. Lower back, elbow and knee WMSDs were both researched in two studies and mean prevalence values of 34% (range 29–39%; SD 7%), 29% (range 9–47%; SD 27%) and 27% (range 15–38%; SD 16%), respectively, were obtained [36, 37].

##### Point-prevalence

De Zwart et al. [27] investigated different secondary industry sectors of which mean point-prevalence values of 16% (range 11–20%; SD 3%), 11% (range 7–15%; SD 3%), 5% (range 4–6%; SD 0%) and 5% (range 3–6%; SD 1%) were obtained for shoulder, neck, wrist/hand and elbow WMSDs, respectively. Kaergaard and Andersen [33] also investigated point-prevalence, however they focused on the textile industry and shoulder/neck WMSDs where a prevalence of 78% was reported.

### Incidence

#### Qualitative analysis

The majority of studies were published between 2000 and 2010 [44–49, 51, 52, 54]. Four countries and ten different industries were obtained. Similar to prevalence data, cohort and cross-sectional study designs were preferred in incidence research with nine [44–50, 52, 54] and two studies [51, 53], respectively. Further, the majority of studies reported a response rate greater than 50% [44, 46–48, 53, 54] and included sample sizes between 100 and 500 participants which is in line with included prevalence studies [48–50, 52–54]. Further, evaluation of WMSDs was primarily performed through a combination of reviewing obtained questionnaire data and conducting physical examinations [48, 51, 52, 54]. All studies reported the employment of a health professional to identify WMSDs [44–49, 51, 52, 54] (Table 3).

**Table 3:** Qualitative overview of incidence of WMSDs in secondary industries of Europe

Author, year, study design, period of measurement, industry & country	Examination method, examination tools & response rate	Demographic characteristics	WSMD & Incidence cases/ Incidence ratio	Overall incidence ratio
**Chen et al. 2006** [44] Cohort: 1996–2001 Food and organic products, petrochemical, metals, and automotive industry – UK	Occupational physicians Not further specified 75% of occupational physicians of OPRA	Not retrievable	Upper limb = 1827/million persons-year; FOM Neck/Back = 792/million persons-year; FOM Lower limb = 121/million persons-year; FOM Upper limb = 1945/million persons-year; PRP Neck/Back = 517/million persons-year; PRP Lower limb = 49/million persons-year; PRP Upper limb = 5618/million persons-year; MAP Neck/Back = 1886/million persons-year; MAP Lower limb = 317/million persons-year; MAP	Could not be calculated
**Cherry et al. 2000** [47] Cohort: 1996–2001 Food and organic products, petrochemical, metals, and automotive industry – UK	Occupational physicians Not further specifie 75% of occupational physicians of OPRA	Not mentioned	Hand/Wrist/Arm = 708; FOM Hand/Wrist/Arm = 960; PRP Hand/Wrist/Arm = 1092; MM Hand/Wrist/Arm = 1164; AM Lumbar Spine/Trunk = 384; FOM Lumbar Spine/Trunk = 276; PRP Lumbar Spine/Trunk = 300; MM Lumbar Spine/Trunk = 684; AM Elbow = 264; FOM Elbow = 348; PRP Elbow = 348; MM Elbow = 516; AM Shoulder = 300; AM	Could not be calculated
**Cherry et al. 2001** [46] Cohort: October 1997–September 2000 Food and organic products, petrochemical, metals, and automotive industry - UK and Scotland	Rheumatologists Not further specified 80% of the society’s membership of rheumatologists.	Not mentioned	Upper Limb = 690; FOM Upper Limb = 144; PRP Upper Limb = 228; MM Upper Limb = 201; AM Lower Limb = 57; FOM Lower Limb = /; PRP Lower Limb = 36; MM Lower Limb = 12; AM Neck/Back = 192; FOM Neck/Back = 51; PRP Neck/Back = 30; MM Neck/Back = 12; AM	Could not be calculated
**Cherry et al. 2002** [45] Cohort: 1996–2001 Food and organic material manufacturing, petrochemical, rubber and plastics manufacturing, metallic and automotive products manufacturing - UK and Scotland	Occupational physicians Not further specified Not mentioned	Not mentioned	Upper limb F = 217/million persons-year; FOM Upper limb F = 342/million persons-year; PRP Upper limb F = 598/million persons-year; MAP Back F = 48/million persons-year; FOM Back F = 172/million persons-year; MAP Upper limb M = 106/million persons-year; FOM Upper limb M = 104/million persons-year; PRP Upper limb M = 443/million persons-year; MAP Back M = 79/million persons-year; FOM Back M = 42/million persons-year; PRP Back M = 153/million persons-year; MAP Lower Limb M = 28/million persons-year; MAP	Could not be calculated
**Descatha et al. 2007** [48] Cohort: 1996–2000 Shoe industry – France	Occupational physician Interview Physical examination 66%	T = 166 T = 66.3%; < 45 years T = 33.7%; ≥ 45 years F = 60.8% M = 39.2%	UWMSD F = 0.238; 1996–1997 M = 0.296; 1996–1997 T < 45y = 0.23; 1996–1997 T ≥ 45 years = 0.33; 1996–1997 F = 0,08; 1997–2000 M = 0.08; 1997–2000 T < 45y = 0.08; 1997–2000 T ≥ 45 years = 0.09; 1997–2000	0.26; 1996–1997 0.08; 1997–2000
**Ha et al. 2009** [51] Cross-sectional: April 2002–April 200 Food and drink, garment, shoe and leather, manufacture of wood and wood products, manufacture of pulp, paper and paper products, publishing, printing and reproduction of recorded media, chemical, manufacture of rubber and plastic products, manufacture of other non-metallic mineral products, manufacture of basic metals, manufacture of fabricated metal products manufacture of machinery and equipment not elsewhere classified, manufacture of electrical and optical equipment, manufacture of motor vehicles, manufacture of other transport equipment, manufacture of furniture and wood, recycling industry – France	Neurologists Occupational physicians Physical examinatio 17.4% of the occupational physicians	F = 113; FDI F = 12; GI F = 28; SLI F = 6; WW F = 12; PPP F = 9; PPR F = 2; CI F = 45; PRP F = 2; NMM F = 6; BM F = 11; FMP F = 26; EON F = 69; EO F = 2; MM F = 2; MOT F = 45; FWI F = 0; R M = 182; FDI M = 1; GI M = 8; SLI M = 24; WW M = 52; PPP M = 17; PPR M = 8; CI M = 84; PRP M = 22; NMM M = 23; BM M = 91; FMP M = 89; EON M = 89; EO M = 63; MM M = 91; MOT M = 57; FWI M = 7; R	Carpal tunnel syndrome F = 38; FDI F = 13; GI F = 30; SLI F = 1; WW F = 1; PPP F = 5; PPR F = 12; CI F = 6; PRP F = 0; NMM F = 3; BM F = 7; FMP F = 0; EON F = 23; EO F = 12; MM F = 0; MOT F = 9; FWI F = 1; R M = 22; FDI M = 1; GI M = 11; SLI M = 6; WW M = 1; PPP M = 2; PPR M = 2; CI M = 5; PRP M = 0; NMM M = 6; BM M = 15; FMP M = 7; EON M = 8; EO M = 10; MM M = 0; MOT M = 9; FWI M = 0; R	Could not be calculated
**Häkkänen et al. 2001** [49] Cohort: January 1987–September 199 Transport industry – Finland	Occupational physicians Physical examination Not mentioned	T = 364 F = 55 M = 309	Upper Limb F = 0.2 Neck/Shoulder F = 0.35 Lower Back F = 0.24 Upper Limb M = 0.11 Neck/Shoulder M = 0.10 Lower Back M = 0.19	Upper limb = 0.13 Neck/Shoulder = 0.14 Lower Back = 0.19
**Leclerc et al. 2001** [52] Cohort: 1993—1994 1996–1997 Electrical, textile, food, and packaging industry – France	Self-report Occupational health physician Self-Administered Questionnaire Physical examination 42.11%	T = 247; EO T = 63; TI T = 143; FI T = 103; P	Carpal Tunnel Syndrome = 0.04; EO Carpal Tunnel Syndrome = 0.06; TI Carpal Tunnel Syndrome = 0.05; FI Carpal Tunnel Syndrome = 0.03; P Lateral Epicondylitis = 0.06; EO Lateral Epicondylitis = 0.03; TI Lateral Epicondylitis = 0.04; FI Lateral Epicondylitis = 0.03; P Wrist Tendinitis = 0.01; EO Wrist Tendinitis = 0.02; TI Wrist Tendinitis = 0.03; FI Wrist Tendinitis = 0.03; P	Could not be calculated
**Monaco et al. 2019** [50] Retrospective cohort: January 2012–December 2015 Transport industry – Italy	Not mentioned Reviewing medical records of health surveillance visits Not mentioned	T = 171 T = 78 (45.6%); 25–34 years T = 60 (35.1%); 35–44 years T = 33 (19.3%); ≥ 45 years F = 14 (8.2%) M = 157 (91.8%)	Upper Limb T = 0.04	0.04
**Ricco and Signorelli 2017** [53] Cross-sectional: January 2012 December 2013 Meat industry – Italy	Trained clinician Full medical assessment Ultrasonography and/or NCS in clinically possible cases 91.8%	T = 434; ȳ = 37.0 years F = 198 (45.6%) M = 236 (54.4%)	Carpal tunnel syndrome F = 0.21 M = 0.11	0.14
**Roquelaure et al. 2002** [54] Cohort: 1996–1997 Shoe industry – France	Occupational physicians Interview Physical examination 90%	Year 1996: T = 253; ȳ = 40.2 years F = 158 (62%) M = 95 (38%) Year 1997: T = 191; ȳ = 41.1 years F = 117 (61%) M = 74 (39%)	Tension Neck Syndrome T = 0.04 (*n* = 7); 1997 Rotator Cuff Syndrome T = 0.06 (*n* = 12); 1997 Medial Epicondylitis T = 0; 1997 Lateral Epicondylitis T = 0.02 (*n* = 4); 1997 Cubital Tunnel Syndrome T = 0.03 (*n* = 5); 1997 Radial Tunnel Syndrome T = 0.01 (*n* = 2); 1997 Carpal Tunnel Syndrome T = 0.12 (*n* = 23); 1997 Guyon’s Canal Syndrome T = 0.005 (n = 1); 1997 Hand-Wrist Tendinitis T = 0.03 (*n* = 6); 1997	Lateral epicondylitis = 0.02 Carpal Tunnel Syndrome = 0.12 Wrist Tendinitis = 0.03

*FOM* Food and organic material manufacture, *PRP* Petrochemical, rubber and plastics manufacture, *MAP* Metallic and automotive products manufacture, *MM* Metals manufacture, *AM* Automotive manufacture, *FDI* Food and drink industry, *GI* Garment industry, *SLI* Shoe and leather industry, *WW* Manufacture of wood and wood products, *PPP* Manufacture of pulp, paper and paper products, *PPR* Publishing, printing and reproduction of recorded media, *CI* Chemical industry, *NMM* Manufacture of other non-metallic mineral products, *BM* Manufacture of basic metals, *FMP* Manufacture of fabricated metal products, *EON* Manufacture of electrical and optical equipment not elsewhere classified, *EO* Manufacture of electrical and optical equipment, *MM* Manufacture of motor vehicles, *MOT* Manufacture of other transport equipment, *FWI* Manufacture of furniture and wood industries, *R* Recycling, *TI* Textile industry; *FI* Food industry, *P* Packaging, *T* Total, *F* Females, *M* Males, *ȳ* Mean age, */ =* Not reported

Upper limb and neck/back disorders were the most commonly studied WMSDs with 11 [44–54] and 6 [44–47, 49, 54] studies, respectively, reporting incidence data. A further discrimination in upper limb WMSDs was made by five studies [47, 51–54] with carpal tunnel syndrome being the most frequently reported upper limb disorder [51–54]. Leclerc et al. [52] and Roquelaure et al. [54] both documented epicondylitis lateralis and wrist tendinitis as upper limb WMSDs. In addition, three studies reported incidence data regarding lower limb disorders without further anatomical details [44–46]. Overall, incidence ratios were reported or calculated for six of the eleven studies [48–50, 52–54]. Calculations could not be performed for other studies due to the lack of sample size descriptions. Overall, incidence ratios of upper limb WMSDs ranged from 0.04 to 0.26 [48–50]. A maximum ratio of 0.14 was reported for carpal tunnel syndrome [53], minimum ratios of 0.12 were stated by Leclerc et al. [52] and Roquelaure et al. [54]. Two authors documented incidence ratios regarding lateral epicondylitis and wrist tendinitis of 0.13 and 0.06, respectively, and 0.02 and 0.03, respectively [52, 54]. Häkkänen et al. [49] stated an overall incidence ratio of 0.19 for back WMSDs and 0.14 for neck/shoulder disorders (Table 3).

## Discussion

The goal of this meta-analysis was to provide insight on the prevalence and incidence of WMSDs in Europe’s secondary industries. Results showed that back (overall), shoulder/neck, neck, shoulder, lower back and wrist WMSDs were the most prevalent with mean values of 60% (range 38–72%), 54% (range 18–83%), 51% (range 32–69%), 50% (range 23–78%), 47% (range 24–71%) and 42% (range 14–64%), respectively. Incidence ratios of upper limb disorders were the most common reported and ranged from 0.04 to 0.26 [48–50]. The food industry was the most prominent researched sector for the prevalence of back (overall), elbow, leg (overall), shoulder, neck, shoulder/neck, and wrist/hand WMSDs subgroups. This specific industry type was frequently associated with high prevalence values of WMSDs [20, 24, 35, 37, 40]. The food sector is often characterised by working in a cold environment, which could form an additional risk factor for developing WMSDs [20, 24, 40].

### Back WMSDs

Overall back WMSDs obtained the highest prevalence values of all subgroups (60%). This finding is corroborated by the European Agency for Safety and Health at Work (EU-OSHA) with a 12-month prevalence value of 55% reported in industrial workers (i.e. plant and machine operators, assemblers), indicating the significant susceptibility of this population for developing back WMSDs [1]. Further, lower back WMSDs were more frequently reported (47%) compared to upper back WMSDs (22%). This corresponds to values reported in the systematic review of Briggs et al. [56] where a 12-month prevalence range between 3 to 55% and medians around 30% were obtained for upper back WMSDs in most occupational groups. This difference between prevalence of lower and upper back WMSDs may explain the growing interest in lower back preventive measures since this anatomical location is clearly more prone to develop WMSDs.

It is assumed that the presence of biomechanical risk factors (e.g. lifting heavy loads or performing repetitive task) and psychosocial risk factors (e.g. low job control or level of support from colleagues) increases the development of WMSDs [2, 57]. This correlation was found in the majority of studies [20, 35, 37, 42]. Therefore, to effectively prevent this type of WMSDs, implementation of robotic devices e.g. exoskeletons or collaborative robots could offer great potential by reducing biomechanical loads. Further, studies implementing the Nordic musculoskeletal questionnaire (NMQ) reported higher prevalence values in the lower and upper back subgroup [22, 26, 31, 35, 37, 42, 43]. It is possible that this examining method provided a more thorough self-evaluation than non-validated questionnaires. However, this was not the case for the overall back WMSDs subgroup. Since other factors could have influenced these results (e.g. characteristics of sample size, presence of risk factors, etc.) and high heterogeneity was obtained for all back WMSDs subgroups, it is recommended to interpret these observations with caution.

Regarding incidence of back WMSDs, Häkkänen et al. [49] reported incidence ratios of 0.19 for males (indicating that over the course of 1 year 19 out of 100 persons reported new back WMSDs) and 0.24 for females working in the transport industry. This indicates a relative limited development of new back WMSDs between 1987 and 1990. A European report of 2005 described relatively similar 1-year incidence ratios of low back WMSDs that ranged between 0.12 and 0.29 for Austria and 0.28 for the Czech Republic [58]. These relatively low incidence values and high prevalence values indicate a limited onset of back WMSDs with long-term complaints indicating the need for effective prevention strategies. Nevertheless, studies researching incidence of (lower) back WMSDs in secondary industries of twenty-first century Europe are scarce and results should be interpreted with caution.

### Upper limb and neck WMSDs

The neck, shoulder, and neck/shoulder subgroup obtained the highest prevalence values of upper limb WMSDs with 51, 50 and 54%, respectively. These findings are in line with the work of Buckle and Devereux [59]. No clear trends in prevalence data could be observed in studies reporting high psychosocial stress or the presence of biomechanical risk factors. Although the exact reason for this observation remains speculative, it would not be surprising to find the cause in the known high heterogeneity. Also no discrepancies were found between studies that obtained prevalence data through validated questionnaires, non-validated questionnaires or expert assessment. An exception was the neck subgroup where the NMQ tends to result in higher prevalence values. It is possible that the thoroughness of this questionnaire led to this observation. However, since only one study in this subgroup refrained from using the NMQ [21], under-representation of other questionnaires could also have attributed to finding this trend. The need for effective prevention strategies is again highlighted in these subgroups. Implementation of new robotic devices to optimise employee’s ergonomics and therefore reduce risks for developing WMSDs form an extremely promising strategy to combat WMSDs in the future [12, 13].

Incidence of upper limb WMSDs was researched the most. Between 1987 and 1990, incidence ratios of 0.13 were reported (indicating that over the course of 1 year 13 out of 100 persons reported new upper limb WMSDs) [49]. Later from 1996 to 1997 and from 1997 to 2000, incidence ratios of 0.26 and 0.8 were obtained, respectively [48]. These results carefully suggest that the occurrence of new upper limb WMSDs decreases over time in secondary industries. However, some caution when interpreting these results is advised since no other studies were included to support these statements. Monaco et al. [50] reported an incidence ratio of 0.04 between 2012 and 2015, implying a further decline of upper limb WMSDs. This is contradicted by data provided by EU-OSHA stating that a significant reduction in upper limb WMSDs incidence data is still absent over the beginning course of the twenty-first century [58]. Lower incidence ratios are consistently reported compared to their prevalence values, indicating that - although the onset of new WMSDs is relatively limited - employees experience long-term chronic disorders, leading high prevalence numbers. This hypothesis is strengthened by the finding of Gold et al. [60] who states that employees affected by upper limb WMSDs were still experiencing the same disorder at least 1 year later.

More specific examples of chronic WMSDs were research by Leclerc et al. [52] who reported incidence ratios of 0.12, 0.13 and 0.06 for carpal tunnel syndrome, lateral epicondylitis and wrist tendinopathy, respectively. In accordance with Leclerc et al. [52], other included studies found similar incidence ratios of 0.14 and 0.12 for carpal tunnel syndrome, 0.02 for lateral epicondylitis and 0.03 for wrist tendinopathy [53, 54]. Notably, incidence values for these specific WMSDs tend to have higher consensus compared to other WMSDs. Although not similar in all variables, both Leclerc et al. [52] and Roquelaure et al. [54] investigated WMSDs during the same period (between 1996 and 1997) and worked with occupational physicians to obtain incidence data. In addition, carpal tunnel syndrome and lateral epicondylitis are two well-defined diagnoses compared to vague WMSD complaints in a nondelineated anatomical area. This clearly emphasizes the need for more precise definitions of WMSDs as well as standardized study procedures. In contrast to upper limb incidence data, there is a noticeable lack of information regarding neck WMSDs of industrial workers of Europe.

### Lower limb WMSDs

Lower limb WMSDs were less prevalent than back or upper limb WMSDs with 29, 11, 33, 17% obtained for the leg, hip, knee, ankle/feet WMSDs subgroups, respectively. EU-OSHA reported a similar trend with a 12-month prevalence of 29 and 30% lower limb WMSDs in 2010 and 2015, respectively [58]. It is possible that there are fewer risk factors present in an industrial setting for developing lower limb WMSDs compared to risk factors for upper limb WMSDs [2]. However, it is noteworthy that epidemiological research or risk assessments regarding lower limbs is generally under-represented which could mediate this discrepancy. Some contradicting trends in prevalence of WMSDs were found in lower limb subgroups regarding the examination method and the presence of biomechanical and/or psychosocial risk factors. Since prevalence values of WMSDs are not only influenced by abovementioned factors, it is plausible that the high heterogeneity, contributed to this absence of homogenous trends in included subgroups.

Three studies reported incidence rates of lower limb WMSDs, however overall incidence ratios could not be calculated due to the lack of sample size information [44–46]. Chen et al. [44] reported incidence ratios of 0.0121 (indicating that over the course of 1 year 1 out of 100 persons reported new lower limb WMSDs), 0.000049 and 0.0317 in the food, chemical and metallic industry, respectively. An incidence ratio of 0.000028 was obtained by cherry et al. [45] in the metallic industry. Substantially less information regarding lower limb incidence values is available in European reports and scientific literature to support this research. However, the trend of relatively low incidence values and higher prevalence values again indicate that recurrence of lower limb WMSDs is common among industrial workers. Insufficient time to heal or rehabilitation could contribute to these long-term disorders. Strategies that temporarily relieve the employee from hard labour and effective rehabilitation could benefit the healing process and limit recurrence of WMSDs.

### Limitations and future research

It is evident that with the high obtained heterogeneity in the majority of subgroups, the included studies did not utilise uniformity in methodology and reporting strategies. Often, lack of information or discrepancies regarding demographic characteristics of included sample sizes, performed tasks, examining methods and examining periods challenged pooling and interpretation of obtained results. This resulted for the majority of subgroups in a limited number of included studies and considerable heterogeneity that could not be further investigated and challenges the interpretation of obtained results. In order to improve uniformity and generalisation of results, a criteria document for physical examination and standardized questionnaires (e.g. the NMQ) could be used. Further, assessment of risk of bias was performed with the tool as described by Hoy et al. [18] which was the most applicable for included studies. Kane and Shamliyan [61] previously argued that uniformity of this assessment tool is challenged by unclearly defined constructs. Therefore, guidelines for each item of the assessment tool were subjectively expanded and adjusted to this research’s specific subject through discussion between authors. This self-interpreted variant of the initial definitions provided by Hoy et al. [18] could have influenced the risk assessment scores. Finally, only a limited number of studies reporting incidence values could be included due to scarcity in literature. This inhibited profound quantitative calculations of incidence ratios and highlights the need for more qualitative incidence research. Nevertheless, besides the high variability, the obtained high prevalence values, especially in food industries, indicate the need for effective rehabilitation and prevention strategies tailored to the unique characteristics of each industry type.

## Conclusion

This review and meta-analysis aimed to provide an epidemiological overview of WMSDs in in twenty-first century Europe’s secondary industries. Back (overall), shoulder/neck, neck, shoulder, lower back and wrist WMSDs were the most prevalent with mean values of 60% (range 38–72%), 54% (range 18–83%), 51% (range 32–69%),50% (range 23–78%), 47% (range 24–71%) and 42 (range 14–64%), respectively. Upper limb disorders were the most investigated WMSD in incidence studies and obtained incidence ratios from 0.04 to 0.26. Data regarding lower limb and back WMSDs were scarce and incidence ratios could not be calculated. Although the onset of WMSDs in general appears to be limited, high prevalence values indicate long-term complaints. These results should be interpreted with caution due the high heterogeneity in the majority of subgroups. However, this highlights the need for future research in the epidemiology of WMSDs as well as the effectiveness of new prevention strategies.

## Supplementary Information

**Additional file 1.**

## Data Availability

All data generated or analysed during this study are included in this manuscript.

## Abbreviations

WMSDs: Work-related Musculoskeletal Disorders
CDC: Centers for Disease Control and Prevention
EU-OSHA: European Agency for Safety & Health at Work
NMQ: Nordic Musculoskeletal questionnaire
