# Supplementary material for: Prevalence and incidence of work-related musculoskeletal disorders in secondary industries of 21st century Europe: a systematic review and meta-analysis
Source: BMC Musculoskelet Disord. 2021 Aug 31;22:751. doi: 10.1186/s12891-021-04615-9 (PMC8408961; doi:10.1186/s12891-021-04615-9)
Supplement: Supplementary file 1 — Additional file 1. [file 12891_2021_4615_MOESM1_ESM.docx]

**Supplementary material**

**Fig. A** Search strategy for each database


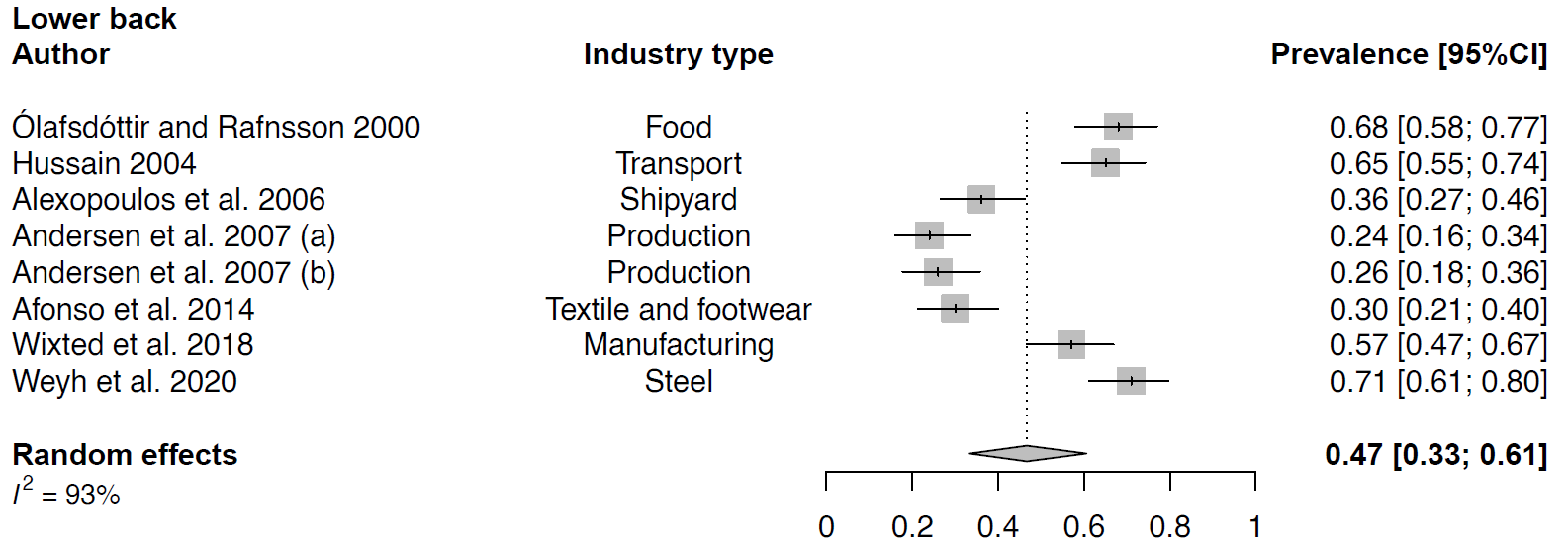


**Fig. B** Meta-analytic overview of prevalence of lower back WMSDs. (a) = baseline; (b) = 24-month follow-up.


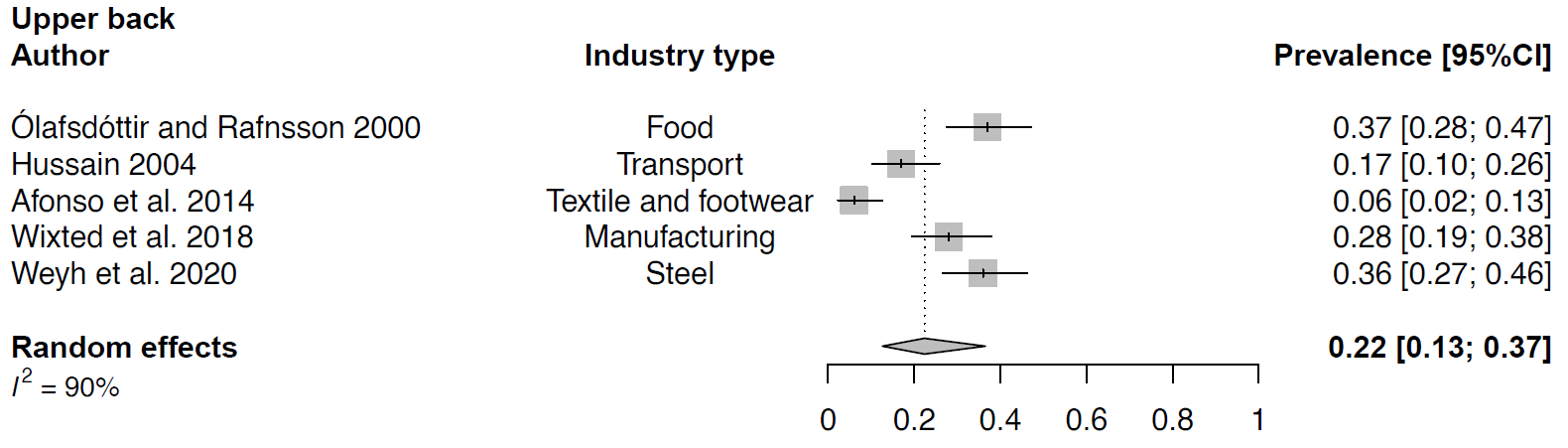


**Fig. C** Meta-analytic overview of prevalence of upper back WMSDs.


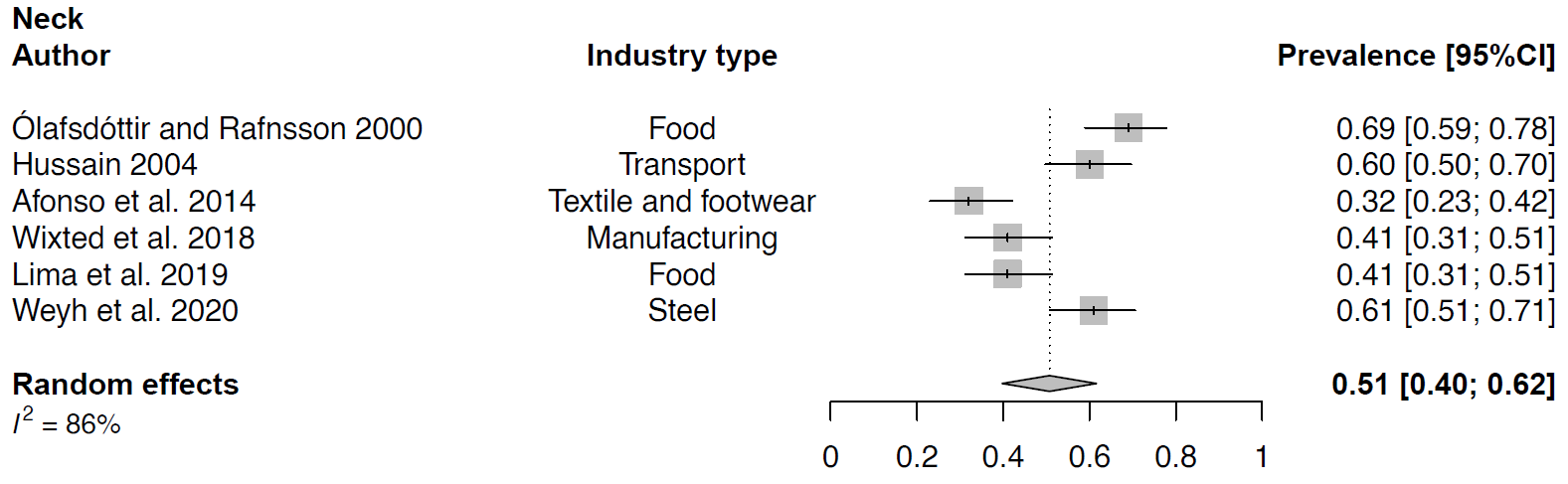


**Fig. D** Meta-analytic overview of prevalence of neck WMSDs.


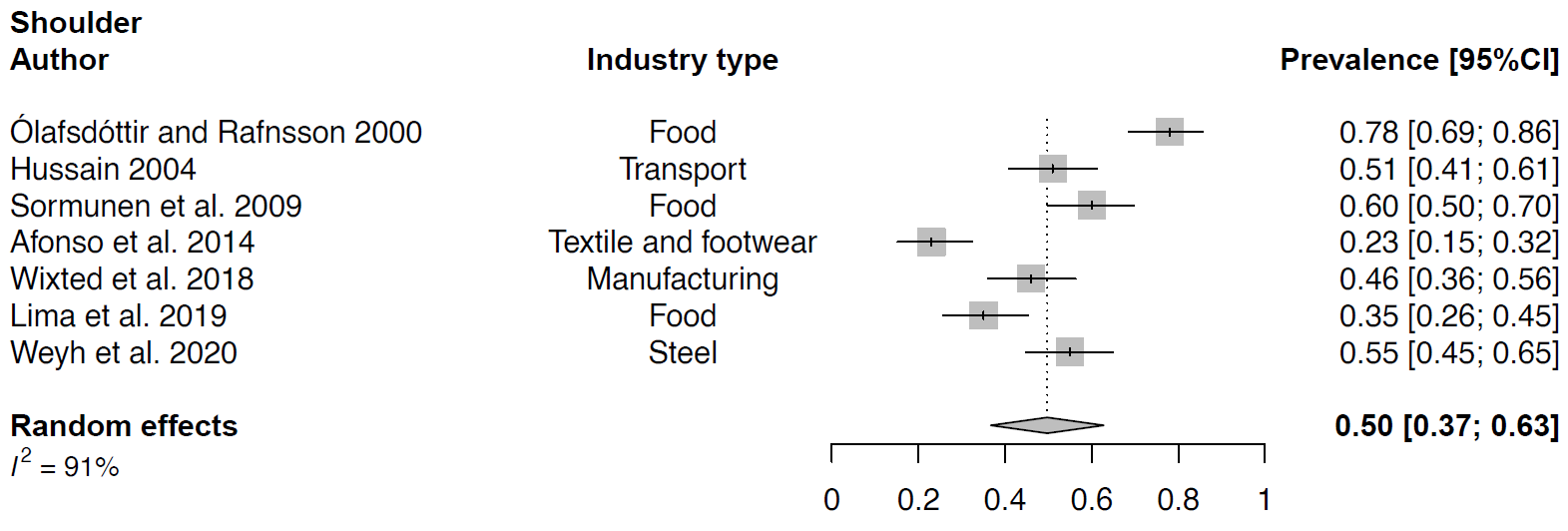


**Fig. E** Meta-analytic overview of prevalence of shoulder WMSDs.


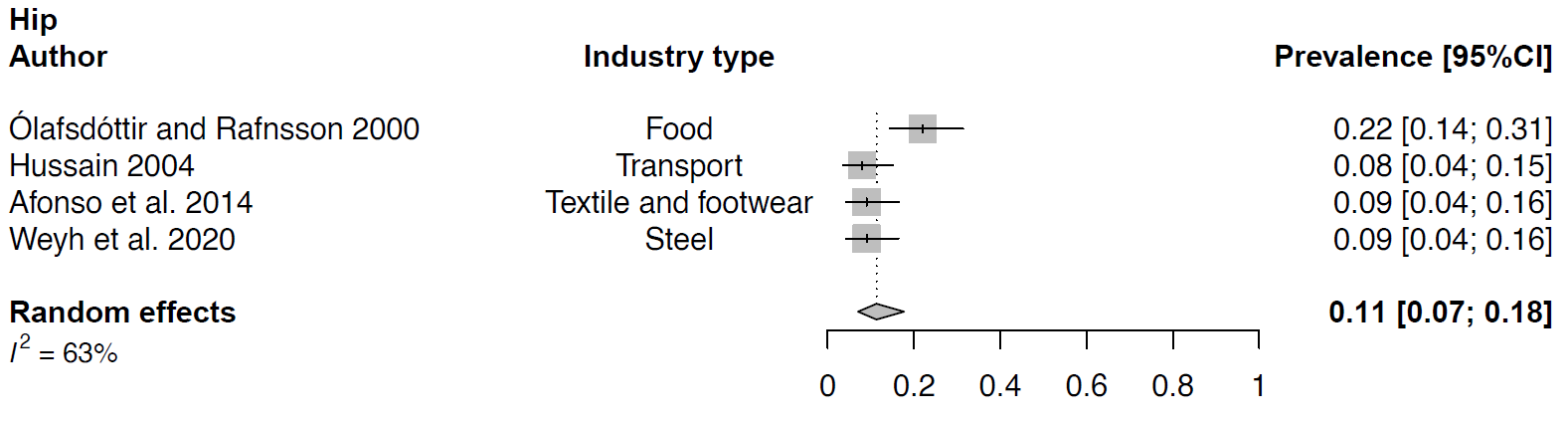


**Fig. F** Meta-analytic overview of prevalence of hip WMSDs.


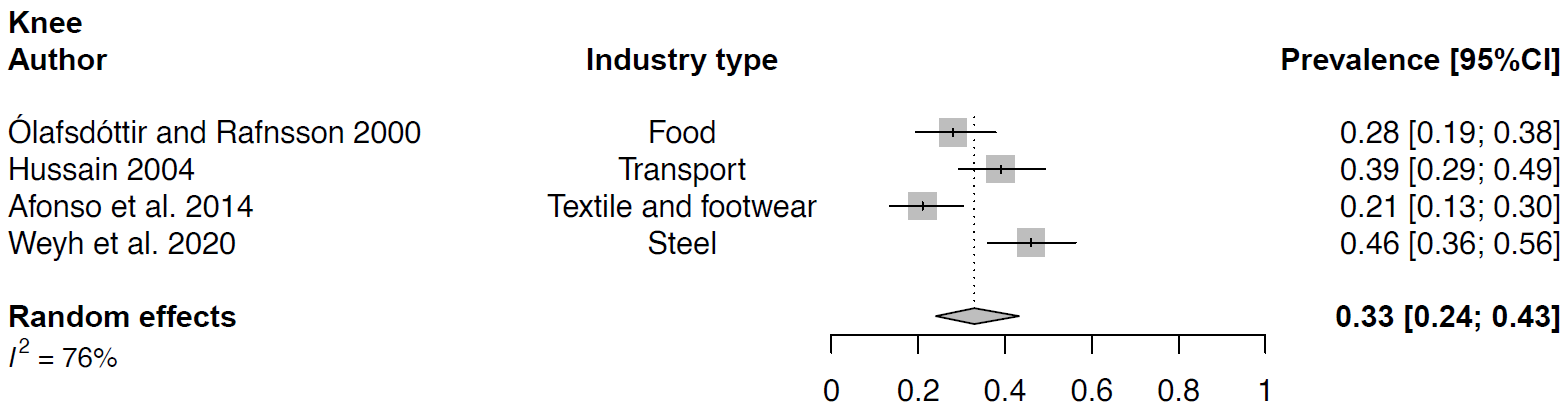


**Fig. G** Meta-analytic overview of prevalence of knee WMSDs.


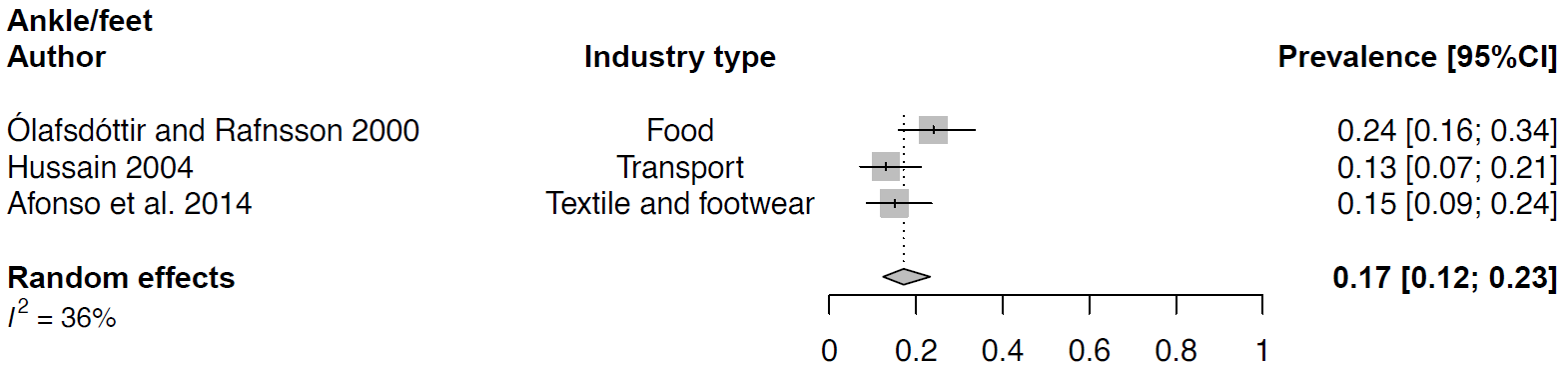


**Fig. H** Meta-analytic overview of prevalence of ankle/feet WMSDs.
